# A Digital Intervention to Alleviate Loneliness and Depression Among Older Persons During the COVID-19 Outbreak

**DOI:** 10.1093/geroni/igab046.3352

**Published:** 2021-12-17

**Authors:** Ella Cohn-Schwartz, Stav Shapira, Daphna Yeshua-Katz, Limor Aharonson-Daniel, A Mark Clarfield, Orly Sarid

**Affiliations:** 1 Ben-Gurion University, Beer-Sheva, HaDarom, Israel; 2 Ben-Gurion University of the Negev, Ben-Gurion University of the Negev, HaDarom, Israel; 3 Ben-Gurion University of the Negev, Beer Sheva, HaDarom, Israel; 4 Ben-Gurion University of the Negev, Beer Shave, HaDarom, Israel

## Abstract

Social distancing has been proven to be effective in reducing infections but may cause ill effects on the mental health of older adults. We evaluated the effects of a short-term virtual group intervention that provided tools to promote better coping, and mitigate adverse mental health effects during the outbreak of the covid-19 pandemic. A Randomized controlled trial tested the effects of a guided intervention comprised of seven online group sessions in which cognitive-behavioral techniques targeting maladaptive beliefs and appraisals were learned and practiced via ZOOM. A total of 82 community-dwelling adults from Israel, aged between 65 - 90 were randomized to either an intervention group (n=64) or a wait-list control group (n=18). Loneliness (UCLA loneliness scale) and depressive symptoms (PHQ-9) were measured pre-intervention, post-intervention, and at 1-month follow-up. The findings showed a significant decrease in loneliness and depression scores in the intervention group with results maintained at 1-month follow-up. There were no significant changes in the wait-list control group. In addition, ten participants (16%) from the intervention group demonstrated a clinically meaningful decrease in depression between baseline and post-intervention, and this was maintained among 7 participants (10%) at 1-month follow-up, compared to only 1 participant (5%) in the control group. Our intervention presents a simple and easy-to-implement tool. Its relevance extends beyond the current pandemic as the skills acquired can be applied in other forms of social crises and during routine life, in order to promote the mental health of older adults who live alone and/or reside in remote areas.

